# The need for a tailored national dementia plan in Ethiopia: A call for action

**DOI:** 10.3389/fneur.2023.1126531

**Published:** 2023-02-28

**Authors:** Biniyam A. Ayele, Seid Ali, Mohammed Anbessie, Yared Z. Zewde, Selam Yoseph, Suzee Lee, Victor Valcour, Bruce Miller

**Affiliations:** ^1^Global Brain Health Institute, University of California, San Francisco, San Francisco, CA, United States; ^2^Global Brain Health Institute, Trinity College Dublin, Dublin, Ireland; ^3^Department of Neurology, College of Health Science, Addis Ababa University, Addis Ababa, Ethiopia; ^4^Amanuel Mental Specialized Hospital, Addis Ababa, Ethiopia; ^5^Department of Psychiatry, College of Health Science, Addis Ababa University, Addis Ababa, Ethiopia; ^6^Memory and Aging Center, University of California, San Francisco, San Francisco, CA, United States

**Keywords:** dementia, non-communicable diseases (NCD), policy, strategies, Ethiopia

## Abstract

Globally, a rapid demographic transition is occurring with a significant increment in the proportion of older individuals. For the first time in history, individuals aged 65 and above outnumber that of children under 5 years of age. In Ethiopia, the life expectancy has shown dramatic improvements in the past few decades and is expected to reach 74 years by mid-century. Older age is considered the most important non-modifiable risk factor for dementia. Likewise, other modifiable diseases such as infectious diseases, non-communicable diseases, particularly cardiovascular diseases, and traumatic brain injuries are associated with dementia. Despite, the high prevalence of dementia risk factors and impending economic and health impact from dementia, no country in the sub-Saharan Africa (SSA), including Ethiopia, has developed a standalone or an integrated national dementia strategic plan to guide the overall effort to improve dementia care in the country. It is vital to design and develop a national dementia plan in line with a framework outlined by the 2017 World Health Organization (WHO) global action plan. The health, social, and economic burden from dementia is expected to be high to the developing countries such as Ethiopia unless clear prevention and management strategies are designed at a national level to cascade the care to the primary care level. The planned strategic policy may focus on improving the knowledge and skills of health care professionals. Translation and cultural adaptation of cognitive, functional, and behavioral assessment batteries is of paramount importance in improving the diagnostic accuracy along with availability of advanced imaging, biomarkers, and dementia treatment.

## Introduction

Globally, population aging is an important issue. According to estimates from the United Nations, the number of people aged 60 or above was 962 million in 2017 and over two-thirds lived in the lower-and middle income countries (LMICs) (1). Dementia is a “brain failure,” can arise from brain dysfunction or disconnection as much as neuronal death, which will result in significant morbidity and mortality (1–3). As a result, individuals experience a wide range of symptoms such as memory loss, visuospatial disorientation, language problems, executive dysfunction, behavioral impairment and more (3–6). Dementia incidence increases with aging and aging is a risk factor for a dementia diagnosis^1^ (7). For the first time in human history, there are now more people on earth older than 65 years than those 5 years and younger, likely due to significant reduction in birth rates and increasing longevity (8). According to WHO, ~60% of individuals with dementia currently live in LMIC and similarly, nearly two-thirds of new cases are expected to occur in these countries (8–10). Like other regions of the world, the population living in the sub-Saharan Africa (SSA) is experiencing a demographic shift. According to a recent estimate, by mid-century the number of people age above 60 years is expected to reach 161 million in sub-Saharan Africa (9, 10).

Ethiopia is the second most populous country in Africa and home to 115 million people. The life expectancy has significantly increased in the past several decades, currently at 67.4 years (11). The expected age of death is estimated to increase by 23% by mid-century. The overall increases in life expectancy are driven by the decline in mortality among those <5 years of age and mortality from common infectious diseases, nutritional deficiency, war, and conflict. Increases in the aging population of Ethiopia will ultimately increase the risk of non-communicable diseases (NCD) such as dementia. Ethiopia and other LMICs are experiencing an epidemiological shift as the number of people suffering from NCD are increasing at an alarming rate and augmenting the existing burden experienced from common communicable diseases such as HIV and tuberculosis. The time is now for Ethiopia to develop a tailored national dementia strategic plan, which will guide the overall dementia care service in the country (8–10, 12).

In 2017 the WHO announced a Global action plan on the public health response to dementia for the year 2017 to 2025 (8). This global action plan was designed to tackle challenges related to the ever-increasing burden of dementia. The global action plan comprises seven action areas including: (a) dementia as a public health priority; (b) awareness & friendliness; (c) risk reduction; (d) dementia diagnosis, treatment, care, and support; (e) support for dementia carers; (f) information system for diagnosis; and (g) research and innovation (8). Despite the ever-increasing aging population and projected increase in the number of individuals living with dementia in the coming decades, as yet, no country in sub-Saharan Africa has established a stand-alone or integrated national dementia strategic plan (9, 13, 14).

In 2019, the Ethiopian Ministry of Health (MoH) published a 5 year second national mental health strategic plan (2020–2025) which focused on menta health, neurological disease, and substance use (15), in order to guide the overall effort to address the challenges in the field. According to the published document from the ministry, the estimated prevalence of dementia in Ethiopia is highly under estimated at 2.4% (15). Contrary to this, Gela et al. (16), reported out of 403 older individuals screened for cognitive impairment, 43.8% of them fulfilled criteria for cognitive impairment. The authors further reported, being older age, unable to read and write, female gender, low income, poor social support, and rural dwelling were an independent predictor of cognitive impairment. Therefore, the proposed national policy should encourage community based epidemiological studies to understand the true magnitude of dementia in the country.

**Figure 1:**
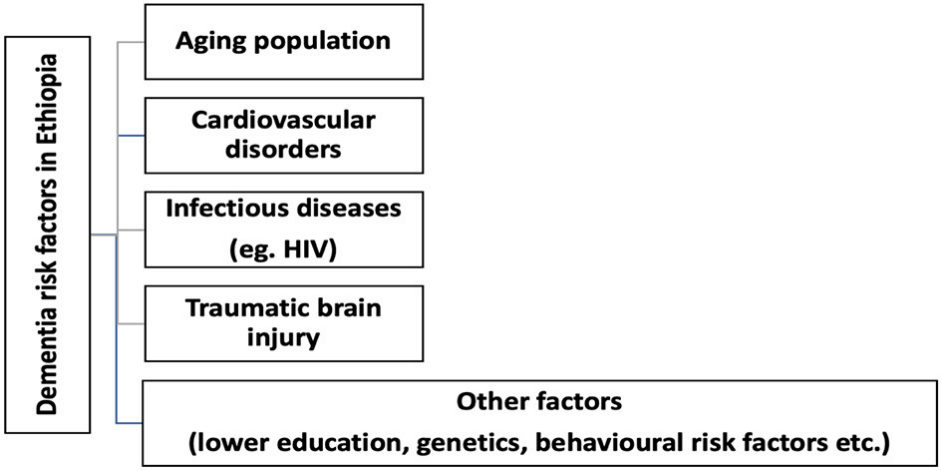
Potential dementia risk factors in Ethiopia.

The country suffers from a high prevalence of diseases known to cause dementia (Figure 1) including HIV infection, malnutrition, NCD, and head injuries known to contribute to dementia and many of whom are modifiable if tailored preventions are implemented (9, 10, 15). These factors add importance for sub-Saharan Africa countries including Ethiopia to develop a tailored and stand-alone or integrated national dementia strategy to tackle the immense economic and health burden dementia will cause in the coming decades. The present call for action was framed in line with the seven action areas included in the 2017 WHO Global action plan with the aims to address opportunities and challenges while providing recommendations and defining the role of potential stalkholders in implementing dementia care in Ethiopia.

## Goal 1: Dementia as a public health priority action

In Ethiopia, the burden of dementia is expected to rise in the coming decades. This is due to the quadruple disease burden the country is currently facing; including the ever increasing aging population due to the increase in life expectancy, the lingering presence of infectious diseases such as HIV, the growing frequency of NCD, and traumatic injuries, each directly or indirectly contributing to the expected surge in dementia (15). It is important for the public, researchers, and policy makers to understand the future burden, to develop, strengthen and implement national strategies and policies, and to develop action plans that address dementia (8, 15). The diagnosis of dementia syndromes such as Alzihmer's disease and Related Dementia (ADRD) is associated with vulnerability, including, high risk of losing their basic human rights, wishes, and preferences (8). Because of this, it is vital to design a tailored national policy which includes protection mechanisms for aging populations in the country. These should include safeguards for issues related to legal capacity, self-determination, supported decision-making, and power of attorney, and for protection against exploitation and abuse in institutions, community, and at the household level (8).

According to the Ethiopian National Social Protection Policy (NSSP) report published in 2019 (17) individuals age 60 years and above are among the most vulnerable segment of population regarding social, economic, and health service (17). In a survey completed by HelpAge International Ethiopia (18), 75% of survey participants reported having at least one chronic disease and of these, 78% reported undergoing medical treatment (18). Though NCDs affect aging population of all nations, those in LMICs are at particularly high risk of NCD, as well as AD and other dementias (8, 15, 17, 18). Health care authorities and all stake holders should work hand in hand to include dementia care in their country's list of health priorities and set up a working functional divisions responsible for the implementation a national dementia strategic plan within the health ministry in order to ensure sustainable funding, clear lines of responsibility for strategic planning, implementation, mechanisms for multisectoral collaboration, service evaluation, monitoring and reporting on dementia (8). Data on the direct costs of dementia in Africa are largely non-existent. However, it has been estimated that the cost of dementia in 2015 rep- resented 6.2 billion US dollars for SSA, of which 70% is attributable to the cost of informal care most often provided by relatives and families of people living with dementia (14).

## Goal 2: Dementia awareness and friendliness

People living with dementia face discrimination, stigma, and dependency which in turn contributes to the psychological, social, emotional, and financial impact of dementia on the community at large (19). Stigma toward people living with dementia is a global problem (20). According to a recent systematic review report, stigmatizing attitudes about AD occur in both health-care workers and in the lay public. Particularly, the impact of perceived dementia stigma in health care professionals may have long term consequence such as delayed diagnosis of dementia and less emphasis in active screening of dementia in vulnerable population such as elderly (20). In SSA including Ethiopia, stigmatization is believed to be rooted in the societal belief systems mostly of culture and faith, where traditional health care workers and faith healers don't see dementia as a disease, but as a feature of normal aging or spiritual possession (14, 21). People living with dementia should be actively involved in health promotion and advocacy programs (22). To reverse this threat, key health promoters with a special interest in dementia health promotion should be approached (19, 21). Recently, a civil association called “*Alzheimer's Ethiopia*,” has been founded by a senior Atlantic fellow and co-author of this paper (YZ), to work actively on raising awareness and informing the public about dementia through different training programs for health care professionals and dementia care givers and media personalities.

## Goal 3: Dementia risk reduction

### Aging population

Advanced age is a well-recognized non-modifiable risk factor for dementia. In the SSA countries the proportion of older people aged 60+ is projected to reach 161 million by 2050 (9, 13). In Ethiopia, over the past three decades life expectancy has increased from 45 to 67.4 years, and the life expectancy of the Ethiopians at birth is expected to reach 74 years by the mid-century (9, 13, 23–25). According to a recent report from the 2019 Ethiopia Mini Demographic and Health Survey (EMDHS), individuals age 65 or older account for 4% of the population (23). The population demography in Ethiopia is largely dominated by a younger population. However, the proportion of aging population is on the rise. For this reason, we need to have a comprehensive and tailored national dementia prevention and care plan to absorb the health and economic burden such devastating diseases would incur in the future.

### Infectious diseases

In the pre-combination of antiretroviral therapy (cART) era, a significant proportion of individuals infected with HIV subsequently developed some degree of cognitive impairment. Some progressed to a more severe form of HIV dementia. However, the post cART era is characterized by a steady and static nature of cognitive impairment leading to mild and moderate subtypes of HIV-associated Neurocognitive Disorder (HAND) (26). These changes are due to improved access to HIV treatment which results in increased life expectancy for people living with HIV (PLWH). However, HIV infection continues to be a major public health issue and a top cause of mortality in Ethiopia (27). A meta-analysis published in 2021 (26) found the pooled prevalence of asymptomatic HAND in Ethiopia to be 39.2% (26). People living with HIV are at higher risk of suffering from other pathogens causing central nervous system (CNS) infection such as neurosyphilis, tuberculous meningitis, and neurotoxoplasmosis, which will further adds risk for cognitive impairment (28–30). Therefore, the national dementia prevention and care strategy should include regular health education on dementia and HIV, early screening of dementia patients for HIV infection, and continuous capacity building training for the health care professionals working at all levels of health care system to prevent and treat potentially preventable causes of dementia such as HAND.

### Non-communicable diseases

According to the Lancet Commission report, 12 modifiable risk factors are responsible for 40% of dementia, globally and over 50% in LMICs (31). The identified contributing factors include less education, hypertension, hearing impairment, smoking, obesity, depression, physical inactivity, diabetes, and low social contact (31). More recently, hearing loss and traumatic head injury were added to the list. Thus, if we implement a well-planned individual or community based dementia prevention strategy, we can reduce the burden of dement substantially, especially in LMIC (31). According to the 2019 DHS survey, more than 90% of adults age above 60 years have not had formal education (23). Considering low education is one of the modifiable risk factors for dementia, the national dementia policy may include strategies to improve access to education for adults. Despite a lingering infectious disease burden in Ethiopia, non-communicable diseases such as hypertension, diabetes mellitus, stroke, and traumatic brain injuries are on the rise (23). In 2014, the WHO estimated that 30% of deaths were due to non-communicable diseases in Ethiopia (23). A review published in 2021 (14), showed that AD and vascular cognitive impairment and dementia remain the most commonly reported dementia phenotypes in Africa (14), which further support the need to adopt a cost effective dementia prevention strategy focusing on a healthy aging and prevention of modifiable risk factors in including stroke (24).

The accumulation of evidence highlights Ethiopia's need for a tailored and focused dementia prevention strategy leveraging the 2017 WHO Global action plan, which comprises the following measures which are potentially protective and can reduce the risk of cognitive decline and dementia (8). The following specific actions could be included in the dementia plan: (a) strengthening regular group/mass exercise including walking or short distance run; (b) discouraging smoking habits through increasing taxes on cigarettes and related products and improving availability of products that's helps individuals quit smoking (e.g., Nicotine patch); (c) routine screening of hypertension and diabetes at the primary health care level and improving referral systems and access to antihypertensive and diabetic medications.

### Traumatic brain injury

According to the Lancet commission report, traumatic brain injury (TBI) contributes 3% of modifiable dementia risk factor (31). There is a paucity of scientific literature on the possible association between TBI and dementia in the SSA including Ethiopia. Nonetheless, considering the high burden of traumatic brain injury and road traffic accidents in the country, TBI could considered as a possible preventable cause of dementia in the country. According to a recently published systematic review, the prevalence of TBI among trauma patients in Ethiopia was 20% (32). Similarly, based on a facility based survey, in Ethiopia, the prevalence of severe TBI ranges from 30 to 40% of all TBI admission (33, 34). Furthermore, according to Ethiopian 2015 STEPwise approach to non-communicable disease risk factor surveillance (NCD STEPS) report (35), two out of 10 respondents were seriously injured as a result of road traffic accidents (35). Considering the health and economic burden on the country, a mitigation strategy is needed that includes public education on the impact of traumatic brain injury on individual cognition and encouragement and strengthening road safety education at the school and community level.

Potential dementia risk factors identified in this review were listed in the Lancet commission report with exception of aging population (31). Furthermore, these risk factors can be addressed by designing an efficient and cost-effective preventive treatment strategy at different age group. For example, according to the report, the presences of lower education, traumatic brain injury, hypertension, diabetes mellitus, and obesity contributes to 35% of the modifiable risk factors. Therefore, the proposed national plan could adopt a cost-effective and easy to implement ways of preventing and managing these dementia risk factors at a national level.

## Goal 4: Dementia diagnosis, treatment, care, and support

Although dementia is often difficult to treat, early detection of the diseases will allow health care providers to plan for an effective management, which will improve quality of life and can help reduce the burden of disease among people living with dementia and their carers (36). Systematic dementia care navigation has also been linked to better quality of life, fewer hospitalizations and decrease costs for governments in HICs. It is vital to train neurologists, psychiatrists, neuropsychologists, geriatricians, and primary care physicians in cognitive neuroscience. To this end, in Ethiopia as in most LMICs, the challenges around dementia prevention and treatment are enormous (14). In Ethiopia, currently there are no formal behavioral neurology training programs or trained behavioral neurologists practicing in the country. As of today, there are a total of 70 neurologist in Ethiopia, in which majority of them are practicing in the major urban cities. Similar problems have been observed regarding the availability and access to advanced neuroimaging modalities such as magnetic resonance imaging (MRI). Likewise, none of the CSF or blood based AD biomarkers such as, β-amyloid (Aβ) peptide and Tau protein (Tau) (37), are available in Ethiopia. The symptomatic treatment options for ADRD include three cholinesterase inhibitors, donepezil, rivastigmine, and galantamine (37), among which, only donepezil is available in Ethiopia.

## Goal 5: Support for dementia carers

With the rising frequency of dementia in LMIC, the burden of caregivers also rises. In Ethiopia there is a paucity of scientific evidence which indicates the magnitude related to the burden experienced by dementia caregivers. A study done in rural Uganda showed that caregivers of people living with dementia experience physical, financial, and psychological stressors (38). Therefore, the proposed dementia plan could include interventions designed to reduce these stressors which will benefit caregivers while improving quality of care for people living with dementia. Furthermore, it is important to tap into the exiting societal support structure such as family members and traditional community-based organizations to have an active involvement in designing dementia support organizations.

## Goal 6: Information systems for diagnosis

The WHO global action plan against dementia, recommends utilization of mobile based or other innovations to improve dementia related knowledge, skills and coping mechanisms in order to facilitate and support the daily lives of people with dementia and their carers (8). According to WHO, using mobile technologies is helpful in improving awareness to bring about change on the key non-communicable diseases risk factors (including tobacco use, alcohol use, unhealthy diet, and lack of physical activity) (39). In Sub-Saharan Africa, 40% of the adult population are now connected to mobile internet services (40). According to 2022 report, nearly 100 million additional mobile subscribers in sub-Saharan Africa by 2025 and Nigeria and Ethiopia will account for almost a third of these (40). Therefore, the national plan should consider using the rapid expansion of mobile and internet service in the country to use it for the purpose of dementia prevention and care.

## Goal 7: Research and innovation

Currently, in Ethiopia, there is a great paucity of scientific studies relevant to dementia care. This likely because of absence of well-trained health care professionals and lack of proper infrastructure to support dementia research in the country. According to the WHO global action plan, the successful implementation of research into dementia aligned with identified research priorities and social and technological innovations can increase the likelihood of effective progress toward better prevention, diagnosis, treatment and care for people living with dementia (8). Thus, the proposed national dementia plan should support establishment of infrastructure to support the dementia research in the country, along with training health care professionals in the field of dementia research.

## Opportunities

In 2017 WHO demonstrated their commitment to reduce dementia burden globally by ratifying a 5 year global action plan response to dementia on the public health (8). Likewise, dementia has been identified as one of top priority neurological diseases in Ethiopia's National Mental Health Strategy documents, indicating the willingness from the government to support the global effort to improve dementia prevention, diagnosis, and care and to further strengthen government commitment to the global call by WHO global dementia action plan (15). Furthermore, it is important to leverage support from different national and international stalkholders who are currently working on improving brain health.

## Conclusion and recommendations

In summary, this a call for action paper recommends developing a tailored stand-alone or an integrated national dementia prevention and care plan which is in congruent with the seven WHO global action plan. We have summarized the recommendations and planned actions in the Table 1.

**Table 1:** Summary of recommendations based on the WHO global action plan (8).

**SN**	**Action areas**	**Recommendations**
1	Dementia as a public health priority	■ Developing a tailored stand-alone or integrated national dementia prevention and care plan ■ If integrated national plan was designed its advisable to integrate dementia care under non-communicable disease, in order not to miss potentially reversible causes of dementia under NCD ■ Assigning a focal person or unit to monitor and report the progress in the dementia care
2	Dementia awareness and friendliness	■ Developing programs that support dementia-awareness campaigns and dementia-friendly programmes that are tailored to the cultural contexts and particular needs of a community ■ Encouraging governmental and non-governmental organizations that's works on dementia advocacy ■ Assigning dementia advocacy ambassadors at a national level
3	Dementia risk reduction	■ Actively identifying potentially reversible causes of dementia risk factors such as cardiovascular diseases, HIV, and traumatic brain injuries ■ Adopting a cost-effective methods of dementia risk reduction is important eg. Mass exercise, encouraging healthy diets, actively surveillance of NCD such as diabetes, hypertension, and heart diseases
4	Dementia diagnosis, treatment, care, and support	■ Improving and strengthening training programs in behavioral neurology ■ Improving dementia diagnostic accuracy through improvement of health infrastructure useful in improving dementia diagnosis e.g., Increasing access to advanced imaging modalities, biomarkers, and culturally adopted neuropsychological batteries ■ Improving access to dementia treatments by including cognitive enhancing drugs to the national drug list
5	Support for dementia carers	■ Designing support mechanism for dementia careers ■ Facilitate access to affordable, evidence-based resources for carers to improve knowledge and skills and reduce emotional stress
6	Information systems for dementia	■ Developing and strengthening facility based and national dementia registries to track the progress of dementia care ■ Leveraging the improvement in the access to internet service by adopting a hybrid care for patients e.g., teleneurology services
7	Dementia research and innovation	■ Mobilizing national and international resources to fund dementia research in the country ■ Utilizing mobile health to reach communities in the rural part of the country

## Author contributions

All authors listed have made a substantial, direct, and intellectual contribution to the work and approved it for publication.

## Footnotes

^1^ www.ebook3000.com
